# Cognitive Effects of Cannabis Use: A Comprehensive Review Across Domains

**DOI:** 10.3390/neurolint17070107

**Published:** 2025-07-15

**Authors:** Andréia Pucinelli de Souza Queiroz, Maria Olivia Pozzolo Pedro, Marcela Waisman Campos, Julio Torales, Antonio Ventriglio, João Mauricio Castaldelli-Maia

**Affiliations:** 1 Department of Neuroscience, Medical School, FMABC University Center, Santo André 09060-870, SP, Brazil; deia.souza.queiroz@gmail.com; 2 Department of Psychiatry, Medical School, University of São Paulo, São Paulo 05403-903, SP, Brazil; maria.oliviapozzolo@usp.br; 3 Department of Cognitive Neurology, Neuropsychiatry, and Neuropsychology, FLENI, Buenos Aires C1428AQK, Argentina; marcelawc@hotmail.com; 4 Grupo de Investigación sobre Epidemiología de los Trastornos Mentales, Facultad de Ciencias Médicas, Psicopatología y Neurociencias Universidad Nacional de Asunción, San Lorenzo 111421, Paraguay; juliotorales@gmail.com; 5 Vicerrectoría de Investigación y Postgrado, Universidad de Los Lagos, Osorno 5290000, Chile; 6 Facultad de Ciencias de la Salud, Universidad Sudamericana, Pedro Juan Caballero 130114, Paraguay; 7 Department of Clinical and Experimental Medicine, University of Foggia, 71122 Foggia, Italy; dr.antonio@gmail.com; 8 Instituto Perdizes (IPER), Hospital das Clinicas HCFMUSP, Faculdade de Medicina, Universidade de Sao Paulo, São Paulo 05021-001, SP, Brazil

**Keywords:** neurocognitive functioning, cannabis-related impairment, residual effects, chronic exposure, abstinence duration

## Abstract

Cannabis is the most widely consumed illicit substance worldwide, with rising use particularly among adolescents and young adults. Accumulating evidence indicates that chronic cannabis use may negatively impact several domains of cognition, yet findings across studies remain varied and fragmented. This comprehensive review synthesizes current knowledge on the long-term cognitive consequences of cannabis use, focusing on attention, executive functioning, learning, memory, language, motor coordination, and social cognition. Consistent impairments have been observed in domains such as attention, executive function, memory, and learning; however, most evidence derives from studies of acute or residual effects. Evidence of long-lasting deficits after extended abstinence remains more limited and methodologically heterogeneous. Acute motor coordination deficits are well established, but persistent impairments in this domain lack conclusive evidence. Effects on language remain inconclusive, and findings regarding social cognition, though limited, suggest potential deficits in emotion recognition and mental state inference. Early onset and high-frequency use are critical risk factors for more severe and enduring cognitive effects. Some deficits may partially reverse with abstinence, although many persist long after cessation. Overall, cannabis use is associated with widespread and lasting cognitive impairments. These findings underscore the need for targeted prevention strategies, especially among youth, and point to future longitudinal and mechanistic research to better understand the nature, persistence, and potential reversibility of these cognitive effects.

## 1. Introduction

Cannabis is an extremely controversial substance that has accompanied humanity for millennia. The earliest record of the plant being smoked dates back approximately 2500 years [1]. The expansion in its use is associated with growing trends of legalization and reduced perception of risk [2,3]. Although cannabis use is less common in Asia, Africa, and Central and South America, annual prevalence across these continents has also been on the rise [4]. However, records of cannabis used for other purposes, such as medicinal and religious purposes and the use of its fiber for fabric production, are even older.

Cannabis contains more than 500 chemical components [5]; among them, the two main compounds are delta-9-tetrahydrocannabinol (Δ9-THC) and cannabidiol (CBD). The former is the primary psychoactive component of cannabis, and the potency of the drug is directly related to its concentration, as are the adverse effects observed after use. The latter, CBD, appears to act similarly to a THC antagonist, showing protective effects against some of the effects of delta-9-tetrahydrocannabinol [6,7]. Cannabis can be found in various forms of preparation: hashish, herbal (flowers and leaves), and oils [8]. Thus, patterns of use vary across the globe. However, a consistent trend in all regions is the increase in cannabis potency in recent years across all preparations. In Europe, for example, herbal cannabis increased in potency from 5.0% in 2006 to 10.2% in 2016, while that of cannabis resin increased from 8.1% to 17.2% [4].

Currently, the World Health Organization (WHO) estimates that there are around 147 million cannabis users worldwide, with an annual prevalence of approximately 2.5% of the global population [9]. According to the WHO, cannabis has shown the largest and fastest growth in consumption since the 1960s, particularly in North America, Western Europe, and Australia. Due to its strong association with youth culture, cannabis use tends to start earlier than with other substances [9]. Additionally, the low cost of the product is directly linked to its high levels of abuse [10]. Cannabis dependence is diagnosed similarly to other substances, using the criteria defined in the International Classification of Diseases (ICD-10) and *Diagnostic and Statistical Manual of Mental Disorders* (DSM-5) classifications [11,12]. Cannabis use is more prevalent among men and young adults, and approximately 10% of users become daily consumers, which is one of the predictors of cannabis use disorder (CUD). The development and severity of CUD are also associated with factors such as genetic susceptibility, psychological disorders, childhood trauma, early onset of use, tobacco smoking, high frequency of use, and consumption of high-potency cannabis [4,13,14,15,16]. This increase in cannabis use has also been accompanied by growing concerns about cannabis-attributable harms, particularly in light of legalization policies. Matheson and Le Foll (2023) [17] reviewed the effects of recreational cannabis legalization and highlighted important sex/gender differences in prevalence, use patterns, and associated harms such as motor vehicle accidents and hospitalizations. Their work underscores the necessity of integrating sex/gender-based analyses into studies on cannabis impacts and suggests that the narrowing gap in use between men and women may be partly driven by legalization trends.

Cannabis use affects human cognition on multiple levels, ranging from basic motor coordination to more complex executive functions [18,19]. Thus, cannabis produces acute, residual, and long-term effects in its users [20,21]. However, the degree of cognitive impairment depends on several factors, including amount consumed, duration of use, frequency, and age of onset [19,22]. Δ9-THC primarily acts on CB1 receptors, which are widely expressed in brain regions involved in cognition, including the prefrontal cortex, hippocampus, and cerebellum. Chronic exposure may induce neuroadaptive changes in these circuits, potentially contributing to lasting cognitive effects. There is substantial evidence of cannabis interference in human cognition, with all cognitive domains being affected to some extent—namely, attention, executive function, learning, memory, language, motor coordination, and social cognition [21]. As cannabis use continues to rise globally, a better understanding of its effects on human cognition becomes increasingly necessary. Several ongoing studies aim to evaluate cognitive function in cannabis users. While previous reviews have focused on acute intoxication or specific cognitive domains, a recent synthesis across all major cognitive domains, incorporating abstinence duration and usage patterns, remains lacking.

The reviews examined the effects of cannabis use across various cognitive domains. Most investigated chronic users, although some focused solely on acute effects. For the purpose of this review, chronic cannabis use was defined as regular (at least weekly) use for at least 12 months. To differentiate the nature of cognitive effects, we classified studies according to abstinence duration at the time of cognitive testing: acute (<24 h), residual (24 h to 7 days), and chronic/persistent (>7 days). Only studies with ≥7 days of abstinence were considered as potential evidence of chronic effects, although select acute/residual studies were retained for comparison. The following sections are structured around seven key cognitive domains: attention, executive function, learning, memory, language, motor coordination, and social cognition.

We conducted a narrative literature review to synthesize the chronic and residual cognitive effects of cannabis use across distinct cognitive domains. The search was carried out between 2021 and 2023 using the PubMed and LILACS databases. We used combinations of the following keywords: “cannabis,” “marijuana,” “cognition,” “cognitive function,” “chronic use,” “residual effects,” along with domain-specific terms such as “memory,” “attention,” “executive function,” “motor coordination,” “social cognition,” “language,” and “learning.” For the purposes of this review, cognitive domains were defined according to standard neuropsychological classifications:

- Attention: The ability to selectively concentrate on relevant stimuli.
- Executive Function: Planning, inhibition, working memory, and cognitive flexibility.
- Learning: The capacity to acquire new information.
- Memory: The processes involved in the storage and retrieval of information.
- Language: Verbal comprehension and production.
- Motor Coordination: Fine and gross motor control.
- Social Cognition: Recognition and interpretation of others’ emotions, intentions, and social signals.

We included studies published in English, Spanish, or Portuguese that investigated chronic or residual effects of cannabis use on human cognition, including both systematic reviews and original empirical research. Studies focused exclusively on acute intoxication or conducted in animal models were excluded, unless they provided relevant comparative insights. Titles and abstracts were screened, followed by full-text assessment of eligible articles. Reference lists of included studies were also reviewed manually to identify additional relevant sources.

This review differs from prior work in several key ways. Unlike Broyd et al. (2016) [21], who synthesized both acute and chronic effects without systematically categorizing studies by abstinence period, our review focuses explicitly on abstinence-defined chronicity. While Figueiredo et al. (2020) [23] provided meta-analytic effect sizes, our task-based narrative synthesis emphasizes methodological nuances across studies. Scott et al. (2018) [22] restricted their analysis to adolescents and young adults, whereas we included studies across the adult lifespan. Lastly, Pocuca et al. (2021) [24] examined older adults in the context of healthy aging, while our review synthesizes evidence across age groups and domains, providing a lifespan perspective on cannabis-related cognitive effects.

**Figure 1:**
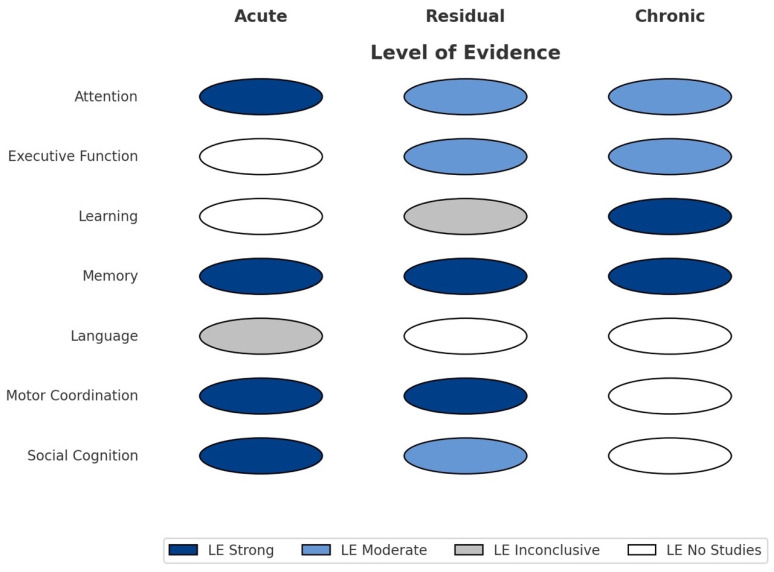
Cannabis-related effects across cognitive domains, categorized by the level of evidence available. Each domain is marked to indicate whether the effects observed in the literature are predominantly acute, residual (i.e., short-term effects after recent use), or chronic (i.e., persistent effects after sustained abstinence).

To visually summarize the patterns of cognitive impact associated with cannabis use, we developed a schematic overview (Figure 1) mapping the primary cognitive domains against the type of effect supported by the literature—acute, residual, or chronic. This figure reflects the heterogeneity of findings across domains, illustrating how certain cognitive functions, such as attention and memory, exhibit impairments across all levels of exposure, while others, like language or motor coordination, are predominantly linked to acute or residual effects. This mapping reinforces the importance of standardizing abstinence criteria and cognitive testing protocols in future research.

Table 1 summarizes the characteristics and main findings of the main studies included in this comprehensive review.

## 2. Attention

Several attentional processes are impaired by chronic cannabis use. Deficits in selective and divided attention have been shown to be related to the duration, frequency, and age of onset of cannabis consumption [19]. Thus, it is evident that users with a longer history of use have greater difficulty identifying irrelevant information. Moreover, early age of onset is considered a strong influence on the development of attention deficits in adulthood, even among light users [44].

The impact of chronic cannabis use on attentional domains has been investigated in several studies included in this review. Although some studies focused exclusively on the acute effects of the substance, there is evidence of chronic impairments in sustained and selective attention among frequent users. Messinis et al. (2006) [36] (chronic) identified significant deficits in sustained and alternating attention tasks in chronic users, even after a period of abstinence, suggesting lasting effects on these domains. Similarly, Gruber et al. (2012) [31] (chronic) observed that individuals who began cannabis use at an early age showed poorer performance on tasks requiring executive attention compared to late-onset users and healthy controls. For example, Anderson et al. (2010) [26] (acute), using the Stroop and UFOV tasks (Table 1), demonstrated acute impairments in divided and selective attention following cannabis administration. On the other hand, Hartley et al. (2019) [32] (residual) assessed occasional and chronic users in a simulated driving environment and found greater impairment in divided attention among chronic users, especially in tasks that required quick responses to simultaneous stimuli.

Böcker et al. (2010) [28] (acute) analyzed event-related potential (ERP) components and identified changes in selective attention among users exposed to cannabis rich in Δ9-THC. However, the study was limited to acute effects, with no assessment of long-term consequences. Finally, Fried et al. (2002) [30] (chronic/residual) reported associations between regular cannabis use and lower performance in composite cognitive tasks that included attention, although they did not isolate the attentional domain specifically.

While attention deficits are among the most frequently reported cognitive consequences of cannabis use, impairments in higher-order executive functions—such as planning, cognitive flexibility, and inhibitory control—may represent a distinct and potentially more persistent pattern of dysfunction. The next section explores evidence of such executive impairments.

## 3. Executive Function

Executive function appears to be diminished in cannabis users, particularly among those who began using the substance at an early age and who consume it frequently [45]. Cognitive flexibility, an executive function related to the ability to shift strategies according to changes in the surrounding environment, is found to be impaired in chronic cannabis users [46].

Several studies have reported impairments in executive functioning among chronic cannabis users. Frequent users show significantly poorer performance on tasks that require planning, reasoning, and inhibitory control, as identified by Valdiviezo et al. (2020) [40] (residual), even after adjusting for confounding variables such as educational attainment and socioeconomic status. These deficits were especially pronounced in verbal fluency tests and tasks involving cognitive flexibility. Messinis et al. (2006) [36] (chronic) observed significant impairments in planning ability and working memory among long-term users, as evidenced by poorer performance on the Wisconsin Card Sorting Test (WCST) and verbal learning tasks. Similarly, Gruber et al. (2012) [31] (chronic) reported that individuals who initiated cannabis use before the age of 16 exhibited more pronounced deficits in executive function compared to those who started later.

Impairments were detected in tasks such as the Wisconsin Card Sorting Test (WCST), semantic classification, and the Iowa Gambling Task (IGT), which assess cognitive flexibility and decision-making under risk (see Table 1). The study by Whitlow et al. (2004) [43] (residual) demonstrated impaired decision-making in heavy users, who were more likely to make disadvantageous choices in IGT.

## 4. Learning

Regarding learning, cannabis users exhibit a general decline in learning processes [47]. Studies have shown that individuals who use cannabis demonstrate deficits in motor learning, a fundamental skill for adapting to constantly changing environmental situations [48]. In addition, verbal learning is also reduced in frequent cannabis users, a condition that may worsen with continued use or improve with abstinence [49].

Schuster et al. (2018) [39] (residual/chronic) reported significant improvements in verbal learning after 30 days of abstinence, suggesting reversibility. In contrast, Messinis et al. (2006) [36] (chronic) found persistent learning impairments despite abstinence of up to 240 h. Learning impairments were measured using paired-associate tasks and pattern recognition tests, as summarized in Table 1. Evidence suggests that chronic cannabis use can compromise learning capacity, particularly in young users. Schuster et al. (2018) [39] (residual/chronic) demonstrated that adolescents and young adults who abstained from cannabis for one month showed significant improvement in verbal learning tests, suggesting a reversible effect linked to active use. Similarly, Fried et al. (2002) [30] (residual/chronic) reported that both current and former cannabis users performed worse on tasks related to the acquisition of new information compared to non-users, with more pronounced deficits among active users.

Moreover, Messinis et al. (2006) [36] (chronic) found that long-term frequent users had poorer results in tasks requiring paired-associate learning, indicating persistent deficits even in abstinent individuals. These findings were corroborated by Valdiviezo et al. (2020) [40] (residual), who observed impairments in tasks involving pattern learning and problem-solving among young male cannabis users. These data suggest an impairment in the acquisition of new information associated with chronic cannabis use, with evidence that abstinence may partially mitigate these negative effects.

## 5. Memory

Chronically, verbal memory appears to be impaired in long-term cannabis users, who show reduced performance in word learning tasks and exhibit deficits in encoding, storage, manipulation, and retrieval processes. As a result, chronic cannabis users tend to learn and recall fewer words. Working memory, which involves the temporary encoding and manipulation of information, is also impaired in different areas, such as visuospatial and auditory n-back tasks [50]. Memory deficits are assessed using tasks such as RAVLT for verbal memory, DRM for false memory susceptibility, and N-back for working memory (see Table 1). In addition, studies have shown that cannabis users are more likely to experience episodes of false memory, that is, recalling events that did not occur or remembering specific details [51].

Numerous studies have identified memory-related deficits associated with chronic cannabis use. The most reported impairments involve verbal and visual memory, as well as difficulties with the encoding and retrieval of information. One consistent finding concerns impairments in visual working memory. In a controlled study, regular cannabis users performed significantly worse on visual memory tasks compared to controls, even after a period of abstinence, suggesting a residual effect rather than solely an acute one [25] (residual). Verbal encoding impairments have also been observed in chronic users. Ranganathan et al. (2017) [37] (acute) found that users had lower accuracy in learning word lists, although retrieval of previously encoded information remained intact, indicating a specific impact on initial storage processes. Moreover, exposure to Δ9-THC has been linked to increased susceptibility to the formation of false memories. False memory effects were measured using the DRM paradigm and VR scenarios, showing increased susceptibility even after short abstinence (Table 1). Kloft et al. (2020) [33] (acute) reported that, even after a prolonged period of abstinence, regular users were more likely to accept incorrect information as true in experimental tasks, which may have important functional implications in clinical and legal contexts.

On the other hand, evidence suggests that prolonged abstinence can lead to improvements in memory performance. In a longitudinal study, Schuster et al. (2018) [39] (residual/chronic) demonstrated that young cannabis users showed significant improvement in both verbal and visual memory after 30 days of supervised abstinence, indicating that some deficits may be reversible.

## 6. Language

Language does not appear to be a cognitive domain significantly affected by cannabis use [52], and some studies even suggest greater verbal fluency among individuals who use cannabis compared to non-users [53]. The reviewed literature indicates that the chronic effects of cannabis use on language remain underexplored and uncertain. Only one older study [41] assessed language function in heavy users, with no significant differences. No recent studies with defined abstinence have focused on this domain. The study conducted by Weckowicz et al. (1977) [41] (acute) assessed cognitive functions in heavy cannabis users, including verbal tasks. The authors found no significant language deficits compared to non-users, although impairments were noted in other domains, such as attention and executive functions [41]. However, methodological limitations, such as small sample size and the study’s publication date, limit the strength of these conclusions.

Thus, the available findings do not allow for a definitive conclusion regarding the existence of chronic language impairments associated with cannabis use, highlighting the need for further studies using specific linguistic instruments to more accurately assess this cognitive domain. It is possible that language function, being more crystallized and less reliant on rapid information processing, may be relatively spared from chronic cannabis-related deficits.

## 7. Motor Coordination

During acute cannabis intoxication, users exhibit reduced motor function performance [28] (acute), and this deficit may persist for some time after chronic cannabis exposure [18] (residual). Cannabis users show slower reaction times and impaired motor control, with evidence indicating a dose-dependent relationship with the amount of THC consumed [54]. Motor coordination was assessed through driving simulation, virtual maze navigation, and reaction time tests (see Table 1).

The chronic effects of cannabis use on motor coordination were assessed in several studies included in this review, with results suggesting impairments in specific situations, particularly among frequent or heavy users. However, the distinction between acute and chronic effects is not always clearly defined in the investigations. The study conducted by Ramaekers et al. (2006) [18] (residual) evaluated motor performance in chronic cannabis users and demonstrated that, even after the acute effects had subsided, subtle alterations in motor coordination and reaction time persisted. These impairments correlated directly with serum THC levels and poorer performance in psychomotor tests. These findings are consistent with those of Weinstein et al. (2008) [42] (residual), who reported worse performance on motor coordination tests among regular users, beyond the acute intoxication phase. On the other hand, Hartley et al. (2019) [32] (residual) observed that chronic users, despite showing some tolerance to the acute effects of THC on vigilance and simulated driving, still exhibited an increased risk of accidents during tasks requiring fine motor coordination and sustained attention.

These findings suggest that chronic cannabis use may be associated with persistent, although subtle, deficits in motor coordination and psychomotor control, raising concerns for activities that demand high levels of motor precision, such as driving and operating machinery.

## 8. Social Cognition

Social cognition remains among the least studied domains in chronic cannabis users. Given that participants were under acute THC, the findings from Ballard (2012) [27] (acute) are considered illustrative of short-term effects and are not interpreted as evidence of chronic impairment. Tasks included facial emotion recognition, cause–effect reasoning, and social absurdity detection (Table 1).

Frequent cannabis users show impairments in facial recognition, suggesting deficits in affective processing [55]. However, occasional cannabis users demonstrate enhanced social cognition compared to non-users, while heavy users display social cognitive functioning similar to non-users. Moreover, cannabis use appears to impair emotion recognition, particularly of negative emotions [56]. In addition to recognition impairments, users also display attentional deficits toward emotionally charged stimuli [40] (residual), which may lead to inappropriate processing of context and daily life events and interactions. Although social cognition remains unexplored relative to other cognitive domains, emerging evidence points to specific deficits among regular users. For example, Valdiviezo et al. (2020) [40] assessed social cognition in young men with chronic cannabis use and found significant impairments in tasks related to theory of mind and emotion recognition, including facial emotion recognition, gaze direction interpretation, and inference of mental states [40].

These results suggest that chronic cannabis use may be associated with impairments in the ability to interpret and respond appropriately to social cues, potentially affecting the quality of interpersonal interactions.

## 9. Discussion

The findings of this systematic review reinforce the evidence that chronic cannabis use is associated with cognitive impairments across multiple domains, especially attention, executive function, memory, and learning. These deficits appear more consistently among users with early onset, frequent use patterns, and consumption of preparations with high concentrations of Δ9-tetrahydrocannabinol (THC), the main psychoactive component of the plant.

Sustained and selective attention were consistently impaired in chronic users, with significant functional impacts in tasks requiring prolonged vigilance, such as driving. Attentional deficits were frequently accompanied by alterations in executive functions, including planning, cognitive flexibility, and inhibitory control, suggesting a broader impairment of self-regulation and decision-making processes [31,40]. These findings are compatible with neuroimaging evidence showing structural and functional alterations in the prefrontal cortex and anterior cingulate gyrus in regular cannabis users [57].

Memory, particularly verbal and working memory, is particularly sensitive to the chronic effects of cannabis. Studies point to impairments in the encoding and storage of new information, with increased susceptibility to false memory formation even after prolonged periods of abstinence [33,37]. Although some of these effects may be reversible with cessation of use, as suggested by Schuster et al. (2018) [39], there is evidence of lasting deficits, especially among those who initiated use during adolescence. Functional MRI studies have shown reduced prefrontal activation and hippocampal volume in chronic users, aligning with behavioral findings of executive and memory deficits [57].

In the domain of learning, results converge toward the presence of deficits in both verbal and motor learning, with partial improvement observed after periods of abstinence. This pattern suggests a direct impact of the substance on circuits related to synaptic plasticity, particularly in the hippocampus and ventral striatum, regions rich in cannabinoid type 1 receptors (CB1). Reversibility appears more pronounced in learning and memory, especially among young abstinent users. However, persistent deficits remain in early-onset, long-term users.

Other domains, such as motor coordination and social cognition, have been less well explored, but available data suggest subtle and contextually relevant impairments. For example, chronic users showed poorer performance on psychomotor tests even after abstinence [18], with practical implications for activities requiring precision and quick reaction time. Regarding social cognition, deficits in facial emotion recognition and inference of mental states were observed, possibly related to altered affective processing in frequent users [40,56]. In contrast, the effects on language remain inconclusive, and further investigation with specific instruments and contemporary samples is needed.

From a methodological standpoint, it is important to note that most of the reviewed studies employed cross-sectional designs and convenience samples, limiting causal inference. However, longitudinal and twin studies suggest that cognitive deficits are unlikely to be solely explained by confounding factors such as social context or genetic predisposition [38]. The heterogeneity of samples, assessment tools, and definitions of abstinence also contributes to variations in findings and should be taken into account when interpreting results.

Another relevant point concerns the composition of cannabis used. THC levels have increased significantly in recent decades, while cannabidiol (CBD) has decreased in many recreational preparations [4]. Although CBD has been suggested as neuroprotective, the evidence remains preliminary and requires controlled trials. This shift may be amplifying adverse cognitive effects, reinforcing the need for studies evaluating the modulatory role of CBD.

Finally, the findings of this review have important implications for public policy and prevention strategies, especially those targeting adolescents and young adults. Given the association between early onset of use, high frequency, and greater severity of cognitive deficits, awareness campaigns and prevention programs should consider the potential impact of cannabis on neurodevelopment. Additionally, cannabis use in medical contexts should be carefully evaluated in terms of risk–benefit ratio, particularly in populations vulnerable from a neuropsychological perspective.

Most studies employed cross-sectional designs with convenience samples, limiting causal inference. Furthermore, variations in abstinence verification, cannabis potency, and assessment tools contribute to outcome heterogeneity. In addition, potential confounders such as polydrug use, psychiatric comorbidities, and socioeconomic status were inconsistently controlled across studies. Moreover, a particularly notable gap in the literature is the paucity of longitudinal studies with rigorous control of abstinence. Only a handful of investigations e.g., [30,49] have followed users over time to assess potential reversibility or progression of cognitive deficits. The field would benefit greatly from prospective cohort studies with clearly defined cannabis exposure profiles and extended periods of verified abstinence.

## 10. Conclusions

The data gathered in this review indicate that chronic cannabis use is associated with significant cognitive impairments, particularly in domains such as attention, executive function, memory, and learning. These effects are more pronounced among users with early onset, frequent use, and exposure to high-THC content. Although some deficits appear to be partially reversible with abstinence, others may persist over long periods, affecting individuals’ academic, occupational, and social functioning.

Despite consistent evidence in some domains, important gaps remain in the literature, including a lack of longitudinal studies, underexploration of domains such as language and social cognition, and limited methodological standardization. Furthermore, few studies account for relevant moderating variables, such as the role of cannabidiol (CBD), biological sex, or the presence of psychiatric comorbidities. These findings underscore the need for prevention strategies specifically aimed at adolescents and young adults, given the neurocognitive vulnerability of this age group. They also highlight the importance of a more balanced debate on the risks and benefits of medicinal and recreational cannabis use, grounded in solid scientific evidence.

Although impairments are consistently reported following recent use, the persistence of cognitive deficits after prolonged abstinence is less robustly supported and varies by domain and study design. Future research should prioritize longitudinal studies with strict abstinence verification to determine true chronic effects. In addition, there is a need for further studies with greater methodological rigor to assess the differential impact of THC and CBD concentrations and explore the neurobiological mechanisms underlying the observed deficits. Only through such efforts will it be possible to fully understand the cognitive consequences of chronic cannabis use and to guide evidence-based public policies, clinical practices, and educational strategies. Addressing these gaps will require longitudinal research with standardized cognitive assessments and carefully documented abstinence periods to better characterize the persistence and trajectory of cannabis-related cognitive changes.

## Figures and Tables

**Table 1:** Characteristics and main findings.

First Author (Year)	Sample Characteristics	Study Design and Methods	Abstinence Period	Main Findings
Adam et al. (2020) [25]	N = 23 per experiment Age: Exp. 1 = 23 (SD = 3.6); Exp. 2 = 23.4 (SD = 4.3) Gender: M = F Country: USA	Design: Randomized Controlled Trial Conditions: THC vs. placebo (1-week interval) Administration: Inhaled THC Task: Visual working memory task (stimulus, delay, and recognition test) Measures: Task performance and metacognitive accuracy	≥72 h, confirmed by urine, oral fluid, and breath testing Participant state during testing: Under the acute influence of 7.5 mg or 15 mg oral THC, randomized crossover	Memory—Participants performed significantly worse under THC (fewer correct items). Metacognitive accuracy was impaired (less awareness of own performance).
Anderson et al. (2010) [26]	N = 70 Age: Mean = 20.9 years (SD = 2.9) Gender: M = 35 and F = 35 Country: USA	Design: Randomized Controlled Trial Tasks: Stroop Test, Digit Span, Reaction Time, and UFOV Measures: Selective and divided attention, working memory, and cognitive flexibility	Participants were sober prior to testing; duration not clearly stated Participant state during testing: Under acute cannabis intoxication (2.9% THC) or placebo	Attention—Cannabis significantly increased the time needed to detect stimuli in both selective and divided attention tasks (UFOV). Users had difficulty identifying relevant information in complex visual environments.
Ballard et al. (2012) [27]	N = 25 Age: 18–35 years Gender: Not reported Country: USA	Design: Randomized Controlled Trial Conditions: 7.5 mg oral THC vs. placebo (cross-over) Tasks: Emotional image viewing (positive, neutral, and negative) Measures: Physiological response and subjective ratings	≥12 h, verified by oral drug and breath alcohol testing Participant state during testing: Under acute oral THC (7.5 or 15 mg) or placebo	Social Cognition—THC reduced emotional reactivity to negative images, with participants reporting lower aversion and emotional intensity. No significant differences were observed for positive or neutral images. Participants felt less anxious and stressed under THC.
Böcker et al. (2010) [28]	N = 24 Age: Mean = 22.8 years Gender: M = 12 and F = 12 Country: The Netherlands	Design: Randomized Controlled Trial Conditions: High-THC cannabis (300 µg/kg) vs. Placebo Tasks: Visual selective attention (color and shape discrimination) Measures: Task performance and ERP (P1 and N2 components)	Required before each session (duration not specified), verified by urine drug screen Participant state during testing: Under the acute influence of high-THC cannabis (up to 69.4 mg THC) or placebo	Attention—THC impaired accuracy and reaction speed in visual attention tasks. Significant reductions in the P1 and N2 event-related potential (ERP) components were observed under THC. These early brain responses are associated with attentional resource allocation (P1) and conflict detection or cognitive control (N2), indicating impaired early-stage attentional processing.
Doss et al. (2018) [29]	N = 23 Age: 18–29 yrs Gender: F:M (12:11) Country: USA	Design: Randomized Controlled Trial (double-blind and placebo-controlled) Conditions: THC (15 mg oral capsule) vs. placebo Procedure: Two sessions spaced 48 h apart Phase 1: Participants viewed emotional (positive, negative, or neutral) images and semantically related word lists Phase 2: Memory retrieval tested for emotional memory and false recall Measures: Memory performance (hits and false alarms), emotional valence modulation, and subjective effect ratings	Participants were sober before THC administration, but no exact duration was specified Participant state during testing: Under the acute effect of 15 mg oral THC at the retrieval stage	Memory Cue Recall: THC did not impact correct or high-confidence hits but significantly increased false alarms and high-confidence false alarms. Emotional valence had no effect. DRM Recognition: THC increased false alarms for both critical lures and unrelated items, reducing adjusted hit rates. Correlation: False alarms positively correlated with subjective THC effect ratings, indicating that stronger perceived drug effects were linked to increased susceptibility to false memories.
Fried et al. (2002) [30]	N = 70 Age: 17–20 yrs Gender: Not available Country: USA	Design: Comparative Study Procedure: Longitudinal assessment of IQ and cognitive functions across multiple developmental stages Cannabis Use: Assessed via structured interviews and self-report questionnaires Cognitive Measures: IQ and domain-specific assessments with a focus on attention and working memory	Participants were categorized based on their current and past cannabis use, as verified by self-report and urinalysis: Current users were further divided into the following groups: - Heavy users: ≥5 joints/week - Light users: <5 joints/week Former users: Those who Those who had not used cannabis regularly for at least 3 months Non-users: Those who had never used more than once per week and no use in the past two weeks Importantly, although subjects reported no use on the day of testing, heavy current users were likely still under the residual influence of THC due to the drug’s long half-life	Attention and Memory: Chronic cannabis users showed a significant decline in cognitive performance over time, especially in attention and working memory domains. IQ Decline: Average IQ dropped by approximately 4 points from childhood to adulthood among chronic users, indicating a long-term cognitive impact associated with sustained cannabis use.
Gruber et al. (2012) [31]	N = 34 cannabis users + 28 non-users Age: Users = 22.8 yrs (SD = 6.57); Non-users = 24.3 yrs (SD = 6.64) Gender: Users = 5F:29M; Non-users = 9F:19M Country: USA	Design: Comparative Study Groups: Users divided into early-onset (<16 years) and late-onset (≥16 years) Assessments: Neuropsychological battery evaluating executive functions, including working memory, inhibitory control, cognitive flexibility, and processing speed	Users were required to abstain for at least 12 h before testing, verified by urine samples.	Working Memory: Early-onset users had significantly poorer performance in manipulating and storing information. Inhibitory Control: These users struggled more to suppress impulsive responses, reflecting reduced attentional regulation. Cognitive Flexibility: Lower capacity to adapt to changing rules or shift strategies, impairing decision-making and problem-solving. Processing Speed: Slower response times in early-onset users indicated diminished cognitive efficiency compared to late-onset users.
Hartley et al. (2019) [32]	N = 30 (15 chronic users [CC] and 15 occasional users [OC]) Age: 20–34 years Gender: Male Country: France	Design: Randomized Controlled Trial; Procedure: Double-blind; participants smoked cannabis in doses adjusted to their typical usage levels Tasks: High-precision simulated driving tasks assessing vigilance and accident risk. Measures: THC levels measured via blood and saliva samples, correlated with cognitive and motor performance	Chronic users were required to abstain for at least 12 h prior to the study. Occasional users were required to abstain for at least 48 h before the session.	Motor Coordination—Driving Vigilance: Cannabis significantly reduced vigilance in both groups. Chronic users exhibited a smaller decline, suggesting partial tolerance. Accident Risk: Both groups faced increased crash risk after cannabis use, with occasional users showing greater impairment. Pharmacodynamic Relationship: Higher THC concentrations were strongly associated with cognitive and motor impairments, though occasional users were more affected than chronic users at comparable THC levels.
Kloft et al. (2020) [33]	N = 64 Age: 22.7 yrs (SD = 2.6) Gender: F:M (32:32) Country: Australia and the Netherlands	Design: Randomized Controlled Trial (double-blind, placebo-controlled) Procedure: Participants (occasional cannabis users) attended two sessions, receiving THC in one and a placebo in the other. Memory was assessed immediately and after one week. Tasks: DRM Word Recognition: Test of false recognition of semantically related word lists. Virtual Reality (VR) Fight Scenario: Eyewitness memory after being exposed to misinformation. Virtual Reality Theft Scenario: Perpetrator’s memory distorted by misleading peer testimony	At least 7 days, confirmed by urine drug screening. Participant state during testing: Under the acute influence of THC, administered in a controlled laboratory setting.	Memory—DRM Task: THC increased false recognition of non-presented words, impairing participants’ ability to differentiate between seen and unseen information. VR Fight Scenario: The THC group was more likely to incorporate misleading information into their memory of the event, demonstrating increased susceptibility to misinformation-based false memories. VR Theft Scenario: Cannabis-intoxicated participants were more prone to accepting false testimony as part of their own recollection. Overall Implication: THC exposure significantly heightened the formation of false memories across all three paradigms.
Matheson et al. (2020) [34]	N = 91 Age: 19 to 25 yrs Gender: F = M Country: USA	Design: Randomized Controlled Trial Procedure: Participants were randomly assigned to receive either active cannabis (12.5% THC) or placebo. Assessments: Mood: Standardized mood scales were applied one hour after cannabis use. Cognitive Performance: Memory and other cognitive tests were administered at two timepoints: one hour and 48 h post-consumption	Yes, 12+ h, confirmed by oral and breath tests Participant state during testing: Under acute THC (12.5%) or in residual phase (24 h later)	Memory—Acute Effects: Participants in the THC group showed decreased word retention shortly after use. Residual Effects (48 h): No significant differences in cognitive performance between THC and placebo groups were observed after 48 h. Social Cognition/Mood: Mood: The THC group reported elevated excitement and positive mood shortly after consumption. Additional Effects: Increased sociability, euphoria, and confusion were reported more frequently in the THC group than in the placebo group.
McClure et al. (2015) [35]	N = 70 Age: 15 to 21 yrs Gender: Not available Country: USA	Design: Randomized Controlled Trial Procedure: Cannabis-dependent adolescents were compared to non-user controls. All participants completed both objective neuropsychological memory tests and subjective self-report memory questionnaires	12 to 24 h, based on self-report. Those who appeared intoxicated at screening were excluded from testing.	Memory—Cannabis-using adolescents significantly underestimated their memory impairments when compared to objective test results. Subjective ratings of memory function did not accurately reflect the cognitive deficits observed in standardized assessments.
Messinis et al. (2006) [36]	N = 64 (20 long-term heavy users, 20 short-term heavy users, and 24 controls) Age: 32.7 yrs Gender: F:M = 15:25 Country: Greece	Design: Comparative Study Procedure: Participants were divided into three groups: long-term heavy cannabis users (≥10 years), short-term heavy users (5–9 years), and controls (used cannabis ≤20 times lifetime, none in the past 2 years). All abstained from cannabis for ≥24 h prior to testing. Cognitive domains were assessed using the following: Rey Auditory Verbal Learning Test (RAVLT); Trail Making Test—Part A (TMT-A); Trail Making Test—Part B (TMT-B); Boston Naming Test (BNT) Covariates controlled included age, IQ, education, and depressive symptoms	Participants were required to abstain from cannabis use for at least 24 h prior to testing. The actual abstinence range was from 36 to 240 h, as verified by urine samples collected before and during testing.	Attention/Psychomotor Speed—Both user groups showed slower TMT-A completion than controls. Long-term users were the slowest (*p* = 0.036), followed by short-term users (*p* < 0.001). Executive Function—Both groups had impaired TMT-B performance, indicating reduced mental flexibility. Long-term users were more impaired than controls (*p* = 0.011); short-term users also differed significantly (*p* < 0.001). Memory— Long-term users performed worse than controls on all RAVLT measures, including delayed recall (*p* < 0.001) and recognition (*p* = 0.015). Short-term users showed milder but significant memory deficits (*p* < 0.01). Language—Only long-term users showed reduced BNT performance (*p* = 0.008), suggesting lexical retrieval deficits not observed in the short-term group.
Ramaekers et al. (2006) [18]	N = 20 Age: 19 to 29 yrs Gender: F:M = 6:14 Country: Germany	Design: Randomized Controlled Trial Procedure: Participants were administered varying concentrations of THC, after which blood and saliva samples were collected to determine THC levels.	At least 72 h (3 days) verified through urine and oral fluid testing.	Motor Coordination and Composite Performance—Higher THC concentrations in serum and saliva were directly associated with greater impairment in cognitive and motor functions. Cognitive and motor deterioration increased with THC dose. Recovery varied based on individual THC clearance, with slower declines in THC linked to prolonged deficits.
Ranganathan et al. (2017) [37]	N = 79 (38 in Session 1; 57 in Session 2) Age: Not available Gender: Not available Country: USA	Design: Randomized Controlled Trial Procedure: Participants completed a series of verbal memory tasks under both THC and placebo conditions. Tasks were divided into the following: Encoding phase: Word lists were presented for memorization Retrieval phase: Participants recalled the memorized words after a delay Analysis: Performance was compared across conditions to assess THC effects on encoding vs. retrieval	Participants were sober during testing, but the duration is not specified, confirmed by urine testing Participant state during testing: Under the acute effects of oral THC (15 mg) in a double-blind, placebo-controlled, within-subject design	Memory-Encoding: THC significantly impaired the encoding of new verbal information. Participants remembered fewer words under THC than under the placebo. Retrieval: No significant differences were found in the recall of previously encoded material, indicating that THC primarily disrupts the learning process, not memory retrieval. Additional Findings: Statistical controls for THC metabolism and baseline cognitive function confirmed the robustness of the encoding-specific impairment.
Ross et al. (2021) [38]	N = 2410 twin pairs Age: Not available Gender: Male and female Country: USA	Design: Longitudinal Twin Study Procedure: Cognitive outcomes were compared within twin pairs discordant for cannabis use (one user and one non-user). This design allowed for control of genetic and shared environmental confounding Assessments: Standardized neuropsychological tests were used to evaluate attention, executive function, memory, and information processing speed	There is no experimental manipulation of cannabis use or controlled abstinence period	Attention—Cannabis users showed deficits in sustained and divided attention. They had increased difficulty maintaining focus during repetitive tasks and in switching attention between different stimuli. Executive Function—A slight but statistically significant impairment was observed in cannabis users. Performance was lower on tasks requiring planning, decision-making, and impulse control, indicating reduced capacity for managing complex behaviors. Memory—Cannabis users had significantly reduced short-term memory compared to their non-using co-twins. The deficit was especially evident in tasks that required holding information for short durations.
Schuster et al. (2018) [39]	N = 88 Age: 16 to 25 years Gender: Female–Male = 37:51 Country: USA	Design: Randomized Controlled Trial Groups: MJ-Abst (n = 62): Underwent four weeks of cannabis abstinence using contingency management (CM); MJ-Mon (n = 26): Received non-contingent monitoring with no abstinence requirement, matched for time and attention Assessments: Cognitive testing with a focus on learning and memory, administered across the intervention period	30 days (MJ-Abst group), confirmed by urine toxicology MJ-Abst: abstinent; MJ-Mon: continued cannabis use	Learning—Abstaining from cannabis significantly enhanced the ability to learn new information. This finding supports the idea that cannabis may disrupt academic learning processes in youth. Memory—Participants in the abstinent group demonstrated marked improvements in learning and recall of new information. Memory performance improved most significantly during the first week of abstinence and continued to improve throughout the four-week period.
Valdiviezo et al. (2020) [40]	N = 36 (18 consumers vs. 18 non-consumers) Age: Consumers = 18.94 yrs (SD = 3.4); Non-consumers = 19.22 yrs (SD = 2.5) Gender: Male Country: Mexico	Design: Cross-sectional comparative observational study Assessments: Executive function tasks: card sorting, semantic classification, and metamemory; social cognition tasks: social judgment, absurdity detection, cause-effect reasoning Statistical analysis: Student’s t-test and Spearman correlation with cannabis use duration and dependence scores	24 h before the assessment	Executive Function—Card Sorting: Lower accuracy (*p* = 0.01); more perseverative errors, indicating cognitive rigidity; Semantic Classification: Reduced abstract grouping performance (*p* = 0.03; *p* = 0.002 for abstract reasoning); Metamemory: More errors (*p* = 0.047); impaired self-monitoring and adaptive regulation of behavior. Social Cognition—Judgment: Lower performance (*p* = 0.007); Absurdities: Poorer identification of incongruities (*p* = 0.001); Causes: Weaker cause identification (*p* = 0.034); Consequences: Impaired consequence reasoning (*p* = 0.01). Interpretation: Cannabis users demonstrated significant deficits in executive functioning and social cognition. Difficulties were particularly evident in tasks requiring flexible thinking, behavioral adaptation, and understanding social cues and cause–effect relationships.
Weckowicz et al. (1977) [41]	N = 48 (24 cannabis users and 24 non-users) Age: 22.5 yrs Gender: Male only Country: Canada	Design: Comparative study Assessments: Field Dependence: Embedded Figures Test and Rod-and-Frame Test Selective Attention: Stroop Test and Selective Listening Task Convergent/Divergent Thinking: Wechsler Memory Scale, Guilford Number Facility Test, Letter Finding Test, and Word Association Test Personality and Values: Personality inventories, social values, and aesthetic judgment questionnaires Tests conducted in the laboratory and at home; total testing time ≈ 3 h	Not specified. Participants in the cannabis user group were active heavy users, with a history of daily cannabis use for no less than three years and an average of approximately 1200 uses. Eight participants smoked several times per day. No abstinence period was imposed prior to testing.	Attention—Cannabis users outperformed non-users in both selective attention tasks (Stroop and Selective Listening), indicating greater ability to filter relevant from irrelevant stimuli. The findings suggest enhanced selective attention among regular cannabis users, potentially due to long-term neuroadaptation or cognitive compensation.
Weinstein et al. (2008) [42]	N = 14 regular cannabis users Age: Mean = 27 yrs (SD = 7.45) Gender: 4 females and 10 males Country: Israel	Design: Comparative study (within-subject) Procedures: Two test sessions per participant; one day: smoked 17 mg THC cigarette and one placebo cigarette; other day: smoked 13 mg THC cigarette and one placebo cigarette Assessments: Cognitive/Motor Tests—Virtual maze (motor precision); Wisconsin Card Sorting Test (WCST) for mental flexibility; gambling task (risk-based decision-making); and time and distance estimation Physiological Measures: Heart rate and blood pressure before/after use. Subjective Measures: Questionnaire on intoxication and pleasure (“high”).	24 h before each test session, verified by self-report, and urine drug screens were conducted to confirm recent use history	Motor and Cognition—Motor Skills: 17 mg THC increased collisions in the virtual maze more than 13 mg. Executive Function: Both doses impaired performance on WCST, with more errors under 17 mg, suggesting reduced cognitive flexibility. Decision-Making: 17 mg led to more high-risk choices in the gambling task, indicating riskier decision-making under higher THC. Physiological Effects: Elevated blood pressure and heart rate post-consumption. Subjective Effects: Increased intoxication and pleasure reported with both doses, more pronounced at 17 mg.
Whitlow et al. (2004) [43]	N = 20 (10 chronic cannabis users and 10 non-users) Age: Mean = 28 yrs (users) and 25 yrs (non-users) Gender: 2 females and 8 males in each group Country: USA	Design: Comparative study Procedures: Cannabis users: daily use for at least 5 years Control group: no cannabis use Assessments: Decision-Making: Iowa Gambling Task (IGT); Cognitive Battery: CANTAB tests for recognition memory and cognitive flexibility (set-shifting); and Mental Health: Anxiety and depression questionnaires	Participants in the cannabis user group were required to abstain from cannabis use for at least 12 h before testing. On average, they had abstained for 14.6 ± 3.1 h, with a range from 10 to 18 h. Urine cannabinoid levels were used to verify a decrease in concentration between day 1 and day 2 as confirmation of compliance	Executive Function and Memory Recognition Memory: No significant differences between groups Cognitive Flexibility (Set-Shifting): Cannabis users made more errors on set-shifting, though not statistically significant. This trend suggests potential subtle difficulties in adapting to changing rules or strategies. Limitations: The small sample size may have limited statistical power to detect significant differences.

United States of America (USA); Useful Field of View test (UFOV); Δ9-tetrahydrocannabinol (THC); female-to-male participant ratio (F:M); Deese–Roediger–McDermott recognition test (DRM recognition); Intelligence Quotient (IQ); Trail Making Test—Parts A and B (TMT-A and TMT-B); Marijuana Abstinence group (MJ-Abst); Marijuana Monitoring group (MJ-Mon); Cambridge Neuropsychological Test Automated Battery (CANTAB).
